# New Loves and Old

**Published:** 1891-01-10

**Authors:** 


### Passing Topics.

### NEW LOVES AND OLD.

There are, perhaps, few speeches about institutions and their work which better repay perusal or afford more good texts for hanging sermons upon, than those delivered by Lord Derby. It is not only that he speaks with the weight and authority of one who knows what he is talking about, but he is able to set forth plain truths in a way concise and clear enough to carry immediate conviction. A sentence of his contained in a recent address to a Liverpool audience is especially to be commended to supporters of the established charities at the present time. "People ought to be warned not to let good work, which is now being done, languish and fail because some project has laid hold of the public imagination. Help the novelty if you will and think it right, but do not help it at the cost of old and well-tried institutions."

The advantage of council like this is that it shows at once the speaker's intimate acquaintance with the characteristics of those whom he is addressing at the moment. It might appear to the uninformed as if advice so homely, and with its propriety so self-evident, were uncalled for and superfluous. To tell a man of philanthropic instincts not to take away much needed help from one useful and well-tried organisation in order to give it to some other undertaking whose utility must be taken upon trust, would seem almost tantamount to calling his possession of common-sense into question. We all appreciate without prompting that it is an unsatisfactory proceeding to rob Peter with a view to pay Paul, and it might seem eminently unreasonable to suppose a man of generous instincts capable of a transaction so doubtful. But experience proves that novelty exercises a fascination in things charitable as elsewhere, and there is no old-established institution, be it hospital or what not, but must, from time to time, put up with the withdrawal of a subscription for no better reason than that some brand-new enterprise has proved more attractive.

It appears as though the amount of money forthcoming for charitable purposes was incapable of increase in the ratio of cosmopolitan need. Certain it is that the effect upon the existing institutions of any extraordinary appeal for help is immediate and appreciable. Only let the Lord Mayor lend the countenance of the Mansion House to the demands of a fund of the first magnitude, and the institutional stars suffer relapse at once. They are put out of sight and as a direct consequence they go out of mind.

We have heard it asserted that the quality of benevolence is feminine. We, ourselves, doubt whether philanthropy can boast any property in sex, but we will admit its professors are sometimes fickle. It is hard for our permanent institutions with their fixed work and unceasing responsibilities to see those whose constancy they had counted upon separating themselves from their earlier loves in order to endow with their affections and their goods all and sundry aspirants. "General" Booth's scheme may be wholly admirable; Lord Zetland's and Mr. Balfour's we know to be so. Yet it will be a misfortune if the greater part of the gifts to these must be withdrawn from indispensable institutions already in sore need. By all means, as Lord Derby intimates, cast your smiles all round if you will, but do not turn your back upon old acquaintances that you may more effectually cultivate the new.

Agricola.

